# Critical role of TLR activation in viral replication, persistence, and pathogenicity of Theiler’s virus

**DOI:** 10.3389/fimmu.2023.1167972

**Published:** 2023-04-20

**Authors:** Byung S. Kim

**Affiliations:** Department of Microbiology-Immunology, Northwestern University Feinberg School of Medicine, Chicago, IL, United States

**Keywords:** TLRs, chronic viral infection, inflammation, apoptosis, autoimmunity

## Abstract

Theiler’s murine encephalomyelitis virus (TMEV) establishes persistent viral infections in the central nervous system and induces chronic inflammatory demyelinating disease in susceptible mice. TMEV infects dendritic cells, macrophages, B cells, and glial cells. The state of TLR activation in the host plays a critical role in initial viral replication and persistence. The further activation of TLRs enhances viral replication and persistence, leading to the pathogenicity of TMEV-induced demyelinating disease. Various cytokines are produced via TLRs, and MDA-5 signals linked with NF-κB activation following TMEV infection. In turn, these signals further amplify TMEV replication and the persistence of virus-infected cells. The signals further elevate cytokine production, promoting the development of Th17 responses and preventing cellular apoptosis, which enables viral persistence. Excessive levels of cytokines, particularly IL-6 and IL-1β, facilitate the generation of pathogenic Th17 immune responses to viral antigens and autoantigens, leading to TMEV-induced demyelinating disease. These cytokines, together with TLR2 may prematurely generate functionally deficient CD25-FoxP3+ CD4^+^ T cells, which are subsequently converted to Th17 cells. Furthermore, IL-6 and IL-17 synergistically inhibit the apoptosis of virus-infected cells and the cytolytic function of CD8+ T lymphocytes, prolonging the survival of virus-infected cells. The inhibition of apoptosis leads to the persistent activation of NF-κB and TLRs, which continuously provides an environment of excessive cytokines and consequently promotes autoimmune responses. Persistent or repeated infections of other viruses such as COVID-19 may result in similar continuous TLR activation and cytokine production, leading to autoimmune diseases.

## 1 Theiler’s virus

Theiler’s murine encephalomyelitis virus (TMEV) belongs to the family of picornavirus, possessing a single positive RNA genome (1, 2). The picornavirus family includes a wide range of human pathogens, including rhinoviruses, cardioviruses, enteroviruses, and aphthoviruses, and TMEV belongings to the cardioviral group (3, 4). Two major subgroups of TMEV have been identified. One subgroup including GDVII and FA viruses, causes rapid and fatal encephalitis. Another subgroup including the BeAn8386 and DA strains, is known as Theiler’s original (TO) viruses. The inoculation of TO viruses intracerebrally into susceptible mice results in a biphasic neurological disease (2, 5–9). The early, acute phase displays flaccid limb paralysis and the degeneration of neurons (poliomyelitis). The late phase exhibits chronic, inflammatory demyelination (2). In contrast to the DA strain, the BeAn strain of the TO virus group is known to induce a clinically undetectable early-phase disease, although it results in a severe late-phase demyelinating disease (8, 9).

Viruses such as TMEV have been associated with CNS diseases including multiple sclerosis (10–14). Multiple sclerosis (MS) is a long-lasting autoimmune-mediated disease, resulting in demyelination in the white matter of the central nervous system (15). To understand the underlying pathogenic mechanisms of MS, several virus-induced models have been investigated (5, 7, 16, 17). Among virus-induced models, TMEV-induced demyelinating disease in mice has been extensively investigated because of the similarities in its histopathologic characteristics (2, 5, 18, 19). In addition, Saffold virus is an emerging human Theiler’s virus group, infecting more than 90% of human populations, which implicates potential importance in public health (20–22). MS may therefore involve chronic viral infections associated with the development of pathogenic immune responses reactive to viral and/or self-antigens. To explain virus-induced demyelinating disease in mice, several hypotheses have been proposed. First, “bystander” damage to CNS tissues occurs because of the host immune response against viral determinants (23, 24). Second, autoimmune responses to myelin proteins from damages to the CNS by anti-viral immune responses cause the disease development (19, 25, 26). Third, CNS damage incurred by persistent antiviral and autoimmune responses are induced by chronic over-stimulation via TLRs and other pathogen pattern recognition receptors (19, 27). Defining the underlying pathogenic mechanisms associated with TLR engagement with well-defined disease system like TMEV-induced demyelination has paramount importance in understanding the role of TLRs on viral persistence, cytokine production, and development of pathogenic immune responses, including autoimmune responses.

**Figure 1:**
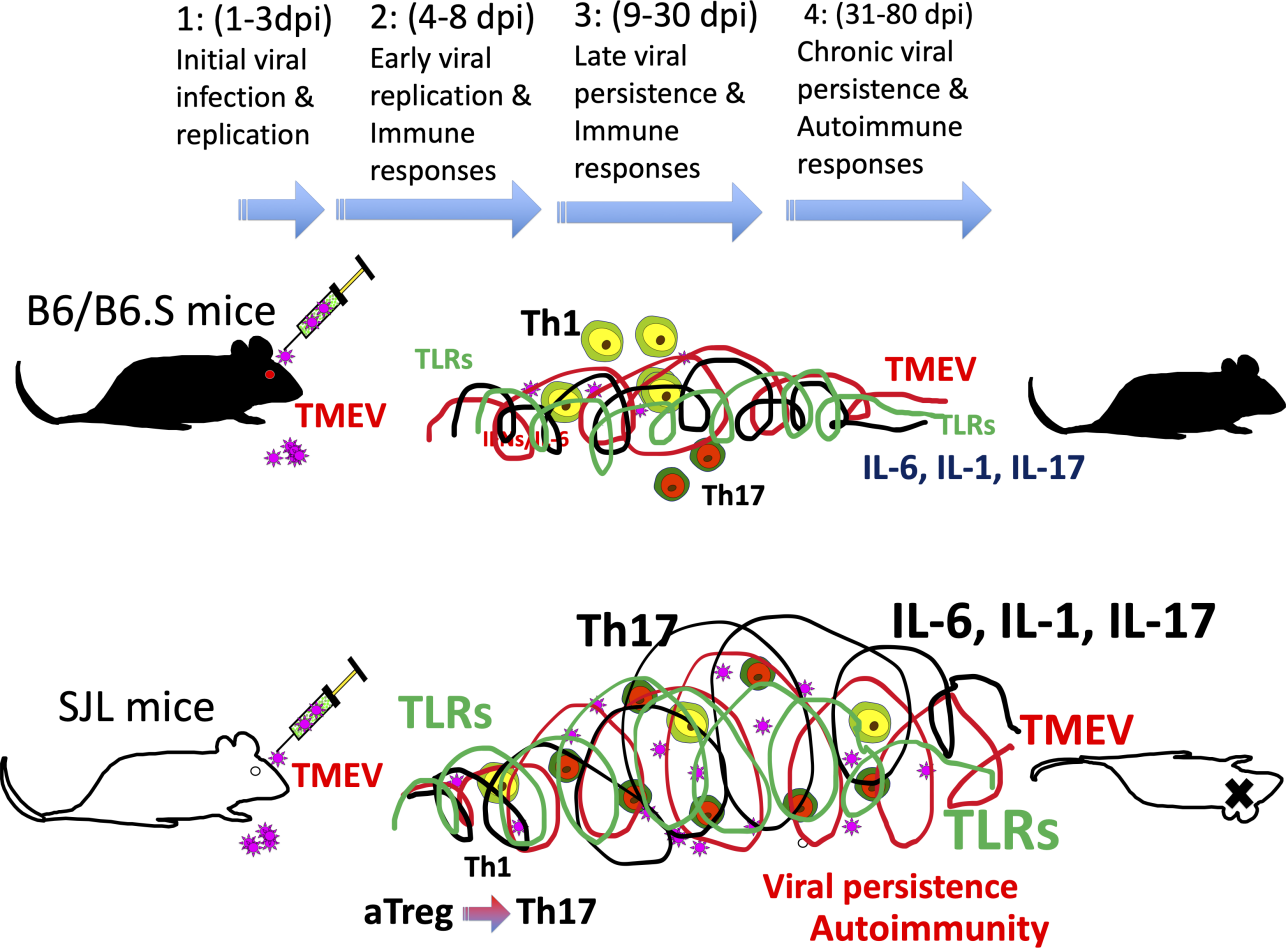
(1) 1-3 d post-infection: initial viral infection; (2) 4-8 d post-infection: early viral infection; (3) 9-30 d post-infection; (4) 31-80 d post-infection: chronic viral persistence. Resistant mice such as B6 and B6.S mice show rapid clearance of virus within 2-3 weeks, whereas susceptible SJL mice fail to clear viral persistence. Consequently, persistent TLR activation throughout infection leads to elevated levels of cytokines such as type I IFNs, IL-6, and IL-1beta, which favors stimulation of pathogenic Th17 response over protective Th1 response. Such pathogenic immune responses lead to the development of demyelinating disease in TMEV-infected SJL mice.

Infection with TMEV causes demyelinating disease only in certain mouse strains. C57BL/6 (B6) mice represent a resistant strain and SJL/J (SJL) mice represent a susceptible strain (28, 29). The viral loads in the spinal cords of resistant C57BL/6 mice are significantly lower than those of susceptible SJL mice throughout the viral infection period (30). Various cell types are permissive to TMEV infection, and these include oligodendrocytes, microglia, and astrocytes in the CNS and the dendritic cells, macrophages, and B cells in the CNS and periphery (31–35). The major cell populations that support viral persistence during chronic TMEV infection are microglia and/or macrophages in the CNS (36–39). Viral replication in microglia from susceptible SJL mice is significantly higher than that in microglia from resistant C57BL/6 mice and the viral load is similarly greater in microglia from SJL mice infected with TMEV compared with microglia from C57BL/6 mice (39). Because TMEV persistence in the CNS is a critically important prerequisite in the pathogenesis of demyelination (30, 40, 41), viral persistence may lead to continuous inflammatory cytokine production and the consequent pathogenic immune responses in the development of TMEV-induced demyelinating disease (**Figure 1**).

## 2 TLR expression and activation status may determine the susceptibility to viral infection

**Figure 2:**
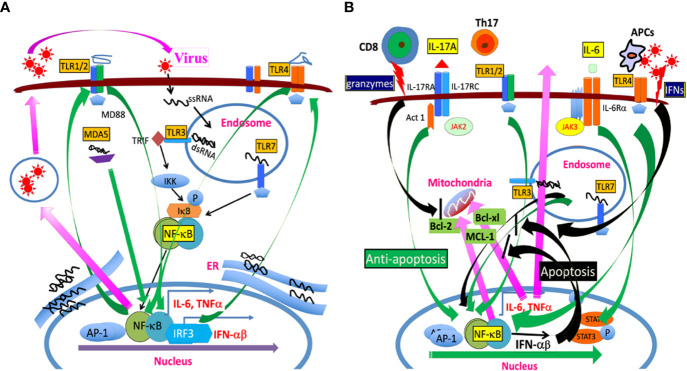
**(A)** TMEV infection releases a single-stranded RNA genome and double-stranded RNA replication intermediate in the endosome. The single-stranded RNA is recognized by TLR7 and the dsRNA intermediate is recognized by TLR3. The TLR signaling activates NF-κB, AP-1, and IRFs, which results in the production of various cytokines such as IL-6, IL-1beta, and type I IFNs. In addition, MDA5 also recognizes dsRNA replication intermediate, leading to NF-κB activation. These activations lead to the additional expression of TLR2/4 and other TLRs which further participates in activating NF-κB. The amplified NF-κB signaling further promotes TMEV replication because TMEV replication is dependent on the presence of activated NF-κB. **(B)** TLR signaling activates NF-κB, which results in the production of anti-apoptotic MCL-1, Bcl-2, Bcl-xL, and IL-6, inhibiting apoptosis induced by cytotoxic T cells and IFNs. IL-6 and IL-1beta further promote the generation of pathogenic Th17 cells. IL-17 produced by Th17 cells together with IL-6 synergistically inhibit the apoptosis of virus-infected cells by activating NF-κB and STAT3, leading to production of additional anti-apoptotic molecules in the mitochondria. The prolonged various TLR signals leading to NF-κB activation participate in the prolonged production of anti-apoptotic molecules and continuous TMEV replication.

Toll-like receptors (TLRs) include a family of members, which recognize microbial products. This family of receptors is known to closely associate with the induction of initial innate immune responses following bacterial and viral infections (42, 43). mRNAs for TLR1–9 are expressed in microglia and stimulation of TLRs on the cells upregulates the expression of MHC class II and costimulatory molecules, which enable the microglia to present antigens to CD4^+^ T cells (38). Activated and/or differentiated cells are far more supportive of TMEV infection/replication (44–46). Consequently, the cytokine production levels in APCs, including microglia, in susceptible SJL mice are higher than those in resistant B6 mice (30, 39). For example, higher levels of TNFα, IL-6 and IL-1β are produced in microglia and macrophages from susceptible SJL mice after TMEV infection, compared with cells from resistant B6, B6.S, or B10.S mice (47–49). Similarly, viral loads in glia and antigen-presenting cells (APCs) from susceptible SJL mice with TMEV-induced demyelinating disease are significantly greater than that in cells from resistant B6.S, B6, and F1(B6XSJL) mice (34, 39). However, the levels of costimulatory molecule expression on APCs from susceptible mice and their ability to stimulate T cells during chronic TMEV infection are significantly poorer, which steers the pathogenic T cell responses (34, 39). Elevated TLR2, 3, 4, and 7 signaling in APCs enhances viral infection and replication (39, 50). Thus, persistent viral infection accompanied by continuous excessive cytokine production leads to viral persistence and the development of pathogenic immune responses. The association of TLR signals with pathogenesis is further supported by the fact that the administration of bacterial lipopolysaccharide (LPS), a ligand for TLR4, or poly I:C, a ligand for TLR3, increases viral loads and elevates the level of inflammatory response (50, 51). Thus, the differences in the type and intensity of TLR signals on antigen-presenting cells may affect the levels of viral infection, replication, and production of IL-1β and IL-6, which are critical factors in the development of demyelinating disease (**Figure 2A**).

## 3 NF-κB activation via TLRs is necessary for TMEV replication and cytokine production

TMEV has a single RNA genome which is recognized by TLR7, and a dsRNA intermediate which is recognized by TLR3 (42, 43). Consequently, TMEV infection leads to activation of NF-κB, AP-1, and IRFs via the TLR signals, resulting in the production of various cytokines, including IL-1β and IL-6 (39, 52–54). The melanoma differentiation-associated gene 5 also recognizes viral messages and participates in the activation of NF-κB (55). The infection of primary glial cells results in the activation of a wide range of chemokine genes, including CXCL1, CXCL2, CXCL10, CCL2, CCL3, CCL4, CCL5, CCL7 and CCL12 (56–58). The production of chemokines and cytokines following various viral infections is dependent on the activation of NF-κB via pattern recognition receptors (32, 52, 59–62). These chemokines further activate CXCR3, CCR7 and CCR5 genes, which further promote the cellular recruitment and infiltration to the CNS (63, 64). In addition, IL-1β, IL-6, IFNα/β, and TNFα produced following TMEV infection further stimulate cells and upregulate the production of CCL2, CCL5, CXCL10 (65–67). These cytokines further promote the development of pathogenic Th17 cells and consequent demyelination (27, 34, 39, 68). Increases in the production of fibrin deposition, adhesion molecules (ICAM and VCAM), and endothelin-1 associated with blood–brain-barrier permeability, contributing to the pathogenesis of demyelinating disease have also been observed after TMEV infection (35, 69–72).

The treatment of resistant C57BL/6 mice with LPS, a ligand of TLR4, or IL-1β increases the viral load in the CNS and leads to the pathogenesis of demyelinating disease (51, 73). Similarly, administration of poly I:C, a ligand of TLR3 leads to the rapid progression of demyelinating disease in mice infected with TMEV (50). The activation of NF-κB via TLRs is associated with TMEV replication and the production of various inflammatory cytokines and chemokine (52, 66, 67). Furthermore, TNF-α, IL-6, and IL-1β produced after TMEV infection further activate NF-κB, promoting increased TMEV replication. NF-κB activation further leads to stimulation of additional TLRs, amplifying pathogenic signals in the deployment of demyelinating disease (45, 52, 66, 74, 75). This is consistent with the fact that TMEV infection and replication are greater in the cells of susceptible mice and that excessive TLR signal and cytokine production, including IL-1β, IL-6, and TGFβ, further promotes the pathogenesis of demyelinating disease (47, 52, 66).

TMEV infection also activates NLRP3 via TLR signaling (74). Activation of NLRP3 inflammasome results in the production of IL-1β and PEG_2,_ promoting the pathogenesis (75, 76). TMEV-infected susceptible SJL mice produce much greater levels of IL-1β and other inflammatory cytokines compared with resistant mice. The presence of high levels of IL-1β and other inflammatory cytokines such as IL-6 promotes pathogenic Th17 responses (47, 68, 73). In addition, IL-17 from Th17 responses further promotes IL-6 production (77, 78), together inhibiting cellular apoptosis (68, 79). The excessive activation of PGE_2_ signaling also contributes to pathogenesis (74, 80) by preventing the T cell killing of target cells (81). TMEV infection also results in the increased expression of PD-1 and PDL-1 via IL-6 signaling, inhibiting cytotoxic T cell function (81, 82). Moreover, the IL-1β signal induces IL-6 production and further promotes Th17 cell expansion (73, 83). Thus, elevated perpetual TLR signaling and consequent persistent cytokine overproduction lead to viral persistence by blocking apoptosis and consequently facilitate pathogenic immune responses in susceptible mice infected with TMEV.

## 4 Excessive cytokine production via TLR activation leads to pathogenic immune responses

TMEV-specific Th1 cells producing IFN-γ can lyse virus-infected cells in a Fas-dependent manner (84). The presence of capsid specific Th1 responses delays the development of demyelinating disease (85–87). In addition, mice deficient in IFN-γ or its receptor genes cannot efficiently clear TMEV infection, resulting in rapid development of demyelinating disease (88, 89). Similarly, deficiency in the IFN-γ receptor significantly accelerates the onset of disease in mice (90, 91). Thus, the level of Th1 responses may play a protective role in the development of TMEV-induced demyelinating disease.

Th17 cells, a subpopulation of Th cells which produce IL-17, are associated with the development of various autoimmune diseases (92–95). Th17 cells are developed in the presence of IL-6, and Th17 cell levels are significantly greater in susceptible mice infected with TMEV. The role of Th17 responses in the pathogenesis of TMEV-induced demyelinating disease has been verified by administrating anti-IL-17 antibodies (68) or using Th17-biased RORγt Tg mice (96). DCs infected with live TMEV producing various innate immune responses, but not epitope peptide-loaded DCs, are able to induce the development of Th17 responses (68). These results indicate that the virus-infected environment, including the presence of various cytokines, is required to induce pathogenic Th17 cells in the development of TMEV-induced demyelinating diseases.

High levels of FoxP3+CD4+ T cells, which constitute a subset of Th cells, appear first in the CNS of virus-infected mice (97, 98). The TLR2-mediated signal induced following TMEV infection may be involved in the early generation of CD25^-^FoxP3^+^CD4^+^ T cells (54, 99, 100). CD25^lo^ FoxP3^+^CD4^+^ T cells are found in levels as much as two-fold higher in the CNS of virus-infected SJL mice compared with B6 mice (101). CD25^lo^FoxP3^+^CD4^+^ T cells do not display a regulatory function (102), and the presence of a high level of CD25^lo^FoxP3^+^CD4^+^ T cells may promote the development of TMEV-induced demyelinating disease (97). CD25^lo^FoxP3^+^CD4^+^ T cells appear to undergo trans-differentiation into Th17 cells following the loss of FoxP3 expression (103, 104). Thus, these CD25^lo^FoxP3^+^CD4^+^ T cells appearing early in the CNS of TMEV-infected mice may be converted into pathogenic Th17 cells in the presence of excessive cytokines (68, 101). Similarly elevated levels of CD25^-^FoxP3^+^CD4^+^ T cells have been detected in patients with chronic hepatitis B virus infection (105) and systemic lupus (106).

Cytotoxic CD8^+^ T cells producing IFN-γ and perforin play an important role in protection of mice from developing TMEV-induced demyelinating disease (107–109). Cytotoxic CD8^+^ T cells recognizing viral determinants appear to participate in causing damages of virus-infected, myelin-producing oligodendrocytes and other cell types in the CNS (110–114). The presence of CD8^+^ T cells is necessary to manifest clinical symptoms, but the protective role of these cells in the development of TMEV-induced demyelinating disease has also been demonstrated (111–114). Thus, certain CD8^+^ T cell populations, depending on their specificity and or cytokine profile are likely to play different roles in the pathogenesis of TMEV-induced demyelination.

TMEV productively infects approximately 50% of primary CD20^+^ B cells and 25% of CD19^+^CD20^+^ cells in susceptible SJL mice (27). TMEV-infected B cells express elevated levels of CD69 as well as MHC class II and costimulatory molecules and exhibit elevated levels of antibody production and enhanced antigen-presenting function to T cells. B cell activation after TMEV infection resembles B cells treated with TLR ligands for TLR2, TLR3, TLR4, TLR7, and TLR9 (27). These results strongly suggest that B cell activation following TMEV infection is associated with TLR signals, consistent with previous findings that various TLR-mediated signals activate B cells (115, 116). TMEV infection also triggers B cells of susceptible mice to produce the excessive production of IFN-α/β, IL-6, IL-1β, and PGE2 (27, 117). These innate immune responses further elevate the activation of B cells to produce antibodies to viral and self-antigens and vigorously promote pathogenic Th17 cell responses (27, 117–119).

B cells producing antibodies to TMEV capsid antigens are detected in the demyelinating lesions and spinal fluids of TMEV-infected mice (120–122). Plasma cells producing anti-TMEV antibodies are also detected in the meninges of CNS parenchyma (123). Antibodies to TMEV determinants play a protective role during the early stage of viral infection (124–126). In the absence of CD8^+^ T cells, antibody response is critically important in protecting mice from Theiler’s virus-induced encephalitis (125). In addition, the pathogenesis of TMEV-induced demyelinating disease is accelerated in mice treated with the monoclonal anti-CD20 antibody (127). However, the contribution of anti-TMEV antibodies to the protection of mice from demyelinating disease is relatively minor compared with the protection by CD4^+^ Th1 and CD8^+^ T cells (125, 128).

## 5 Role of NF-κB activated by TLRs in viral persistence

APCs from susceptible SJL mice are highly susceptible to TMEV infection and the viral infection leads to the activation of NF-κB via TLRs (27, 34, 52). The activation of NF-κB leads to the production of high levels of TNF-α, IL-1β, and IL-6, which are associated with activation of B cells and directional T cell responses (129–131). Type I IFNs produced in consequences of TLR activation contribute to the activation of NF-κB and cellular apoptosis (34, 52, 132, 133). In contrast, high levels of type I or II IFNs provide only transient protection against TMEV infection and thus high levels of these cytokines in susceptible SJL mice may not participate in controlling viral loads in TMEV-infected mice (34, 132, 134). The resistance of TMEV infection against type I IFNs is consistent with previous observations in other viruses (134–136).

Activation of NF-κB is necessary for TMEV replication (45) and consequently the state of host cell activation determines the level of viral replication (137). Following TMEV infection, TLR2, 3, 4, and 7 are associated with the production of various cytokines in different glial cells and professional antigen-presenting cells (27, 39, 52, 54, 138). TLR7 recognizes the single-stranded TMEV RNA genome and TLR3 interacts with the double-stranded RNA replication intermediate in the endosome (48, 52, 139). TMEV infection results in the engagement of various TLRs and other related signals to activate NF-κB, AP-1, and IRFs (54, 55, 140, 141). In turn, these signals result in the production of various cytokines, including TNFα, IL-1β and IL-6. NF-κB activation leads to the elevated expression and activation of TLRs including TLR2/4, which further activate NF-κB at a higher level (54). Such amplified NF-κB signaling further promotes the expression of anti-apoptotic Bcl-2 and Bcl-xL molecules, supporting vigorous TMEV replication (34, 142, 143). Such prevention of apoptosis following TMEV infection may result in further persistent viral replication and excessive cytokine production (**Figure 2B**).

## 6 Production of anti-apoptotic cytokines by virus-infected cells

IL-6 is a major cytokine produced following TMEV and other viral infections and displays an anti-apoptotic function (50, 144, 145). IL-6 inhibits the cytolytic function of CD8^+^ T cells and drives Th17 responses. IL-17 produced by Th17 cells also inhibits cytolytic function of virus-reactive CD8+ T cells, further promoting TMEV persistence (68). Furthermore, IL-6 and IL-17 together synergistically inhibit cytotoxic function of virus-specific CD8^+^ T cells (79). IL-17 is also known to enhance tumor growth in animals, supporting the inhibitory function of IL-17 on cytotoxic CD8+ T cells (146, 147). Cytotoxic CD8+ T cells and some CD4+ T cells induce the cytolysis of virus-infected cells through the granule exocytosis and or the activation of Fas–FasL pathway (86, 148–151). IL-17 upregulates the expression of Bcl-2 and Bcl-xl molecules via the NF-κB pathway and subsequently these prosurvival molecules protect the target cells from the apoptosis induced by cytotoxic CD4+ and CD8+ T cells (68). Nevertheless, the synergistic inhibition of apoptosis of virus-infected cells by IL-17 and IL-6 may serve as a powerful means for viral persistence. The inhibition of apoptosis extends the life of virus-infected cells, resulting in prolonged viral replication and persistence (**Figure 2B**). Thus, the interaction between virus and host cells via IL-17 and IL-6 and/or other cytokines results in persistent viral infection and prolonged harmful immune responses including various autoimmune responses, which ultimately lead to the development of TMEV-induced demyelinating disease (152). Similarly, persistent and or repeated infections of COVID-19 appear to stimulate TLRs, leading to cytokine storms and autoimmune responses in pathogenesis (153–156).

## 7 Viral persistence, continuous TLR signals, and cytokine storms lead to autoimmunity

Chronic infection with TMEV results in antibody responses to self-antigens including myelin basic protein (MBP) in the CNS of infected mice (25, 27, 157). Similarly, CD4^+^ T cell responses to viral antigens and myelin-associated autoantigens, including MBP and proteolipid protein, have been detected during persistent infection with TMEV (19). In addition, a CD8^+^ cytotoxic T cell population, recognizing both viral and self-antigens, has been identified in TMEV-infected SJL mice (158). Thus, various immune responses, including antibody, CD4^+^ T cell, and CD8^+^ T cell responses to viral and CNS autoantigens, are induced in mice with chronic TMEV infection. Sequestered autoantigens to the CNS may be released following TMEV-induced tissue damage, and these autoantigens subsequently induce autoimmune responses under constant elevated TLR stimulation and cytokine production (19, 27). The initial insults to the CNS by T cells reactive to viral antigens appear to be necessary to the pathogenesis of demyelinating disease (84, 113, 159). Such immune-mediated initial tissue damage in addition to virus-induced cellular apoptosis may be necessary to result in the development of TMEV-induced demyelinating disease.

Following intraperitoneal infection of systemic lupus erythematosus-prone NZBWF1 and BXSB male mice with TMEV or Coxsackie virus, the production of autoantibodies to several nuclear autoantigens has been rapidly accelerated (27). Since these mice have either duplicated or altered TLR genes (115, 160–162), the elevated expression of TLRs and their continuous stimulation is likely responsible for autoimmunity. These results strongly suggest that chronic TMEV infection continuously stimulates preexisting autoimmune cells via virus-induced TLR-mediated polyclonal activation. This notion is supported by the fact that the treatment of cells with the ligands of different TLRs activates B cells and T cells similarly to TMEV infection (27). Because virus-reactive T cell responses are required for the development of TMEV-induced demyelinating disease, immune-mediated tissue damage to the CNS may be necessary (68, 107, 111, 159). Therefore, these anti-viral and autoimmune responses appear to participate in protecting and/or damaging the related tissues (19, 68, 159, 163–165). However, the presence of high levels of proinflammatory cytokines during chronic viral infection may lead to the development of harmful autoimmune responses (166–168). Taken together, persistent TMEV infection leads to continuous stimulation of various TLRs and consequently results in TLR-mediated polyclonal activation of B and T cells. Although the roles of autoimmune responses in the pathogenesis of TMEV-induced demyelinating disease are not certain, they are likely to contribute to the overall pathogenic outcome by participating in further tissue damage and/or cytokine production.

## Author contributions

BK: writing—review and editing, funding acquisition.
